# Supplementary material for: Spatiotemporal dynamics of ecto-5′-nucleotidase (CD73) in mouse retina under physiological conditions
Source: Development. 2026 Feb 2;153(2):dev205013. doi: 10.1242/dev.205013 (PMC12912271; doi:10.1242/dev.205013)
Supplement: Supplementary information [file develop-153-205013-s1.pdf]

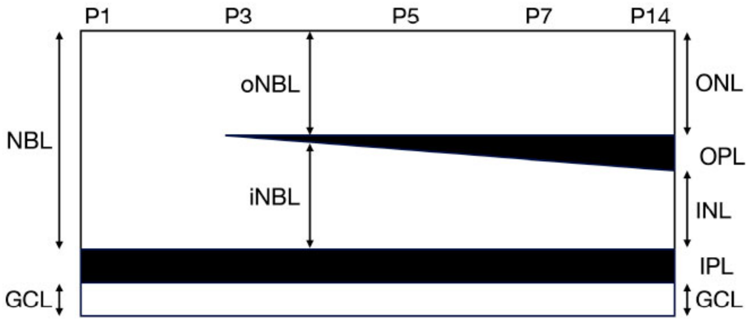

Fig. S1. Retinal layer formation from P1 to P14. This schematic shows the developmental progression of mouse retinal layers from birth (P1) through P14. At P1, the retina comprises two nuclear layers: the GCL and the NBL. By approximately P3, the NBL divides into an outer (oNBL) and inner (iNBL) neuroblastic layer, and the outer plexiform layer (OPL) first becomes detectable. From around P5–P6 onward, the oNBL and iNBL are referred to as the outer nuclear layer (ONL) and the inner nuclear layer (INL). Abbreviations: GCL, ganglion cell layer; iNBL, inner neuroblastic layer; INL, inner nuclear layer; NBL, neuroblastic layer; oNBL, outer neuroblastic layer; ONL, outer nuclear layer; OPL, outer plexiform layer.

Development • Supplementary information

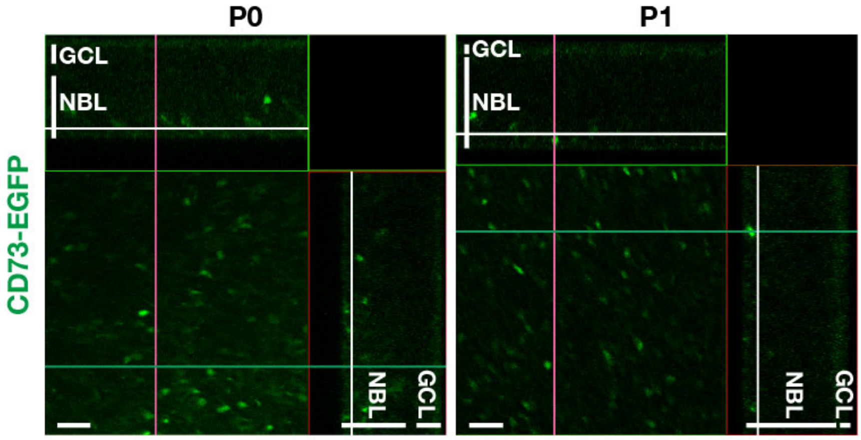

Fig. S2. CD73-EGFP expression pattern at P0 and P1 in the whole-mount retinas. Representative images of whole-mount from *CD73-EGFP* mice at postnatal day 0 (P0, left) and P1 (right). Orthogonal views are shown for the xy plane (center), xz plane (top), and yz plane (right). The white lines in the xz and yz views mark the z-position of the xy plane. The green and magenta lines indicate the y- and x-positions, respectively, which were used to generate the cross-sectional planes. Scale bars: 20 µm.

Development • Supplementary information

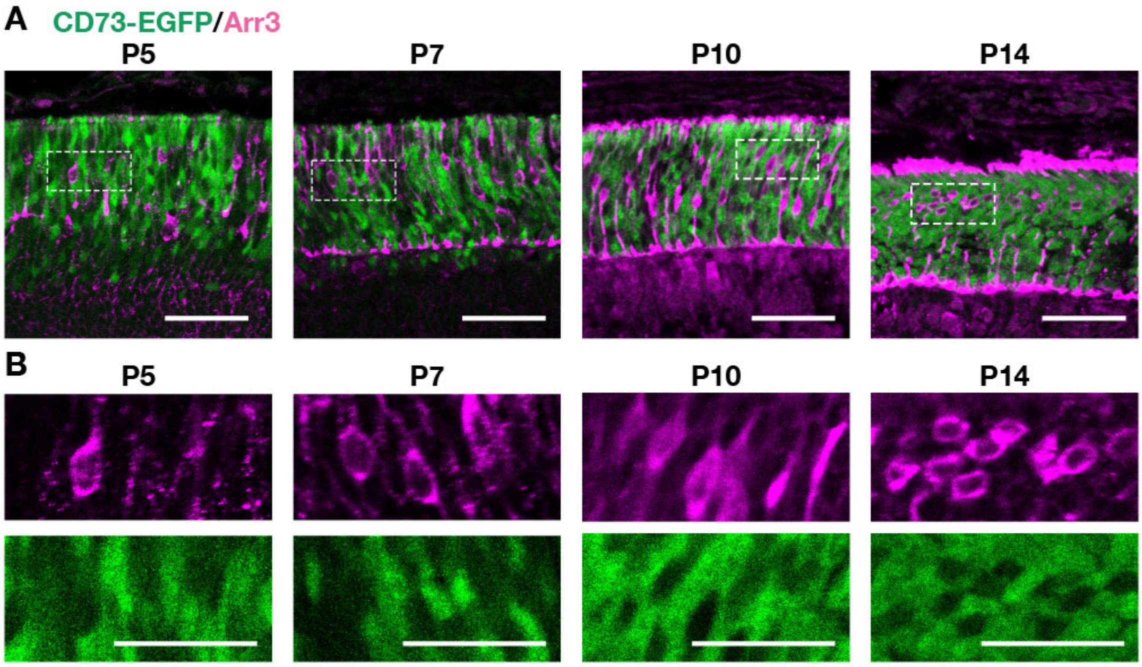

Fig. S3. CD73-EGFP expression is detected in the rod lineage from P5 to P14. (A) Representative images of transverse sections from P5 to P14. Arr3 (cone arrestin, magenta) marks the cone lineage. (B) Higher-magnification view of the dashed box in (A). The top shows the Arr3 (magenta) channel, while the bottom shows the CD73-EGFP (green) channel. Scale bars: 50 µm (A), 25 µm (B).

Development • Supplementary information

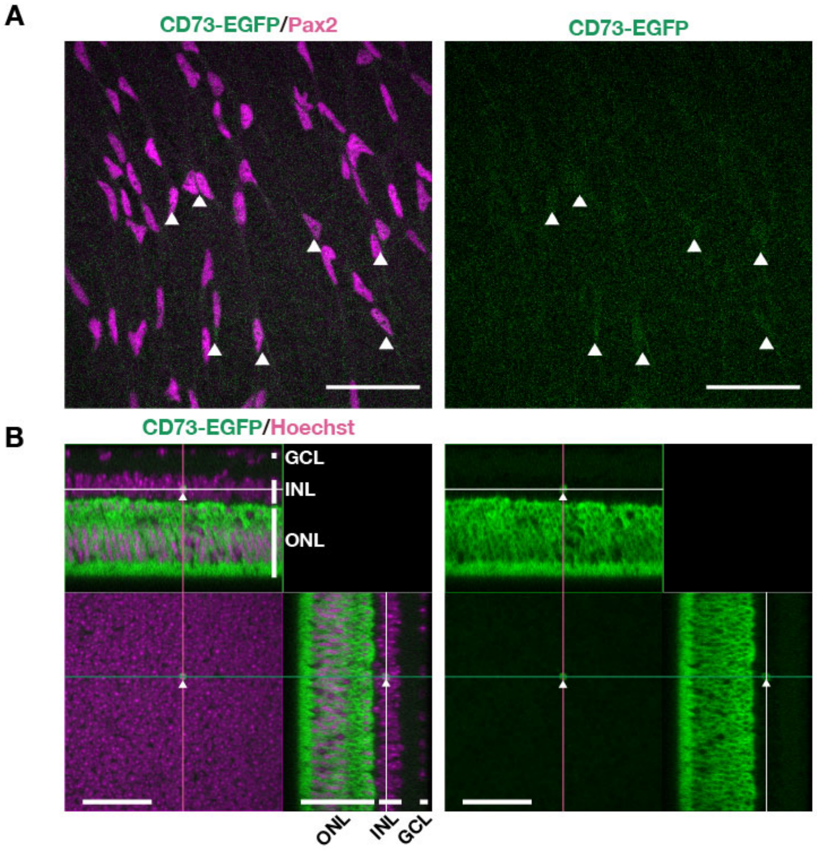

Fig. S4. CD73-EGFP in early postnatal astrocytes and in the adult inner nuclear layer. (A) Representative images of the retinal nerve fiber layer (RNFL) at P1 showing faint CD73-EGFP signals in Pax2^{+} astrocytes. Arrowheads mark low-intensity EGFP signals above local background. Left, merge (CD73-EGFP, green; Pax2, magenta); right, CD73-EGFP single-channel (green). (B) Orthogonal views of representative EGFP^{+} cells in the inner nuclear layer (INL) at 3 months of age. The main panel shows the xy plane; the xz (top) and yz (right) planes are orthogonal slices (green and magenta lines, respectively; white lines in xz and yz indicate the z-position of the xy plane). Arrowheads indicate EGFP^{+} cells in the INL. Left, merge (CD73-EGFP, green; Hoechst, magenta); right, CD73-EGFP single-channel (green). Scale bars: 50 µm. Abbreviations: GCL, ganglion cell layer; INL, inner nuclear layer; ONL, outer nuclear layer.

Development • Supplementary information

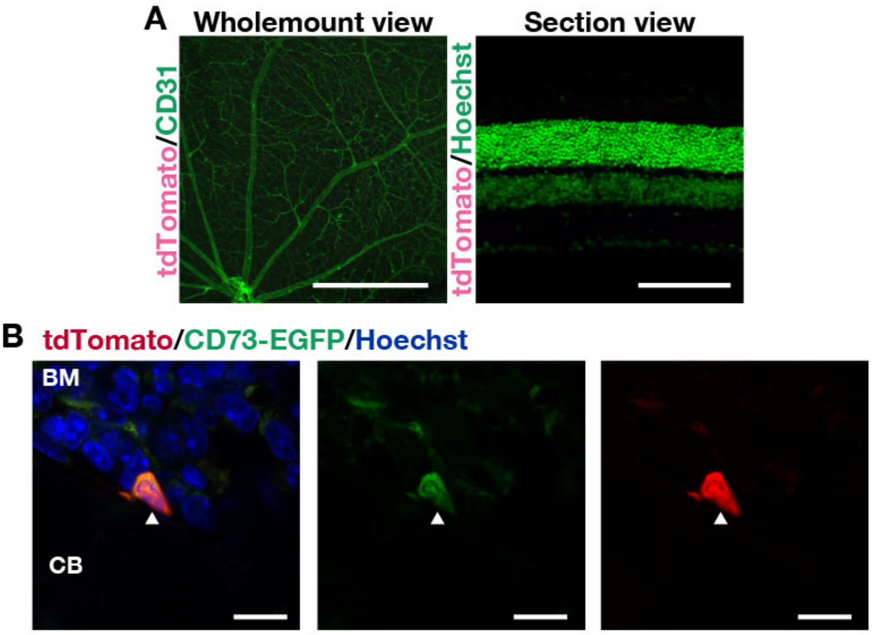

Fig. S5. Basic characteristics of the *CD73-CreER^{T2};tdTomato* mouse line. (A) Representative images of the retinas from 3-month-old *CD73-CreER^{T2}* mice without tamoxifen injection (no-injection control). The left shows a whole-mount view of the retina, and the right is a cross-sectional view. Left: CD31 (green) labels blood vessels. Right: Hoechst (green) labels nuclei. tdTomato is shown in magenta in both panels. (B) Representative femoral bone image from *CD73-EGFP; CD73CreER^{T2};tdTomato* mice treated with tamoxifen at 3 months of age. EGFP^{+} (green)/tdTomato^{+} (red) cell was observed at the inner surface of cortical bone. Hoechst (blue) labels nuclei. Abbreviations: BM, bone marrow; CB, cortical bone. Scale bars: 500 µm (A, left); 100 µm (A, right); 10 µm (B).

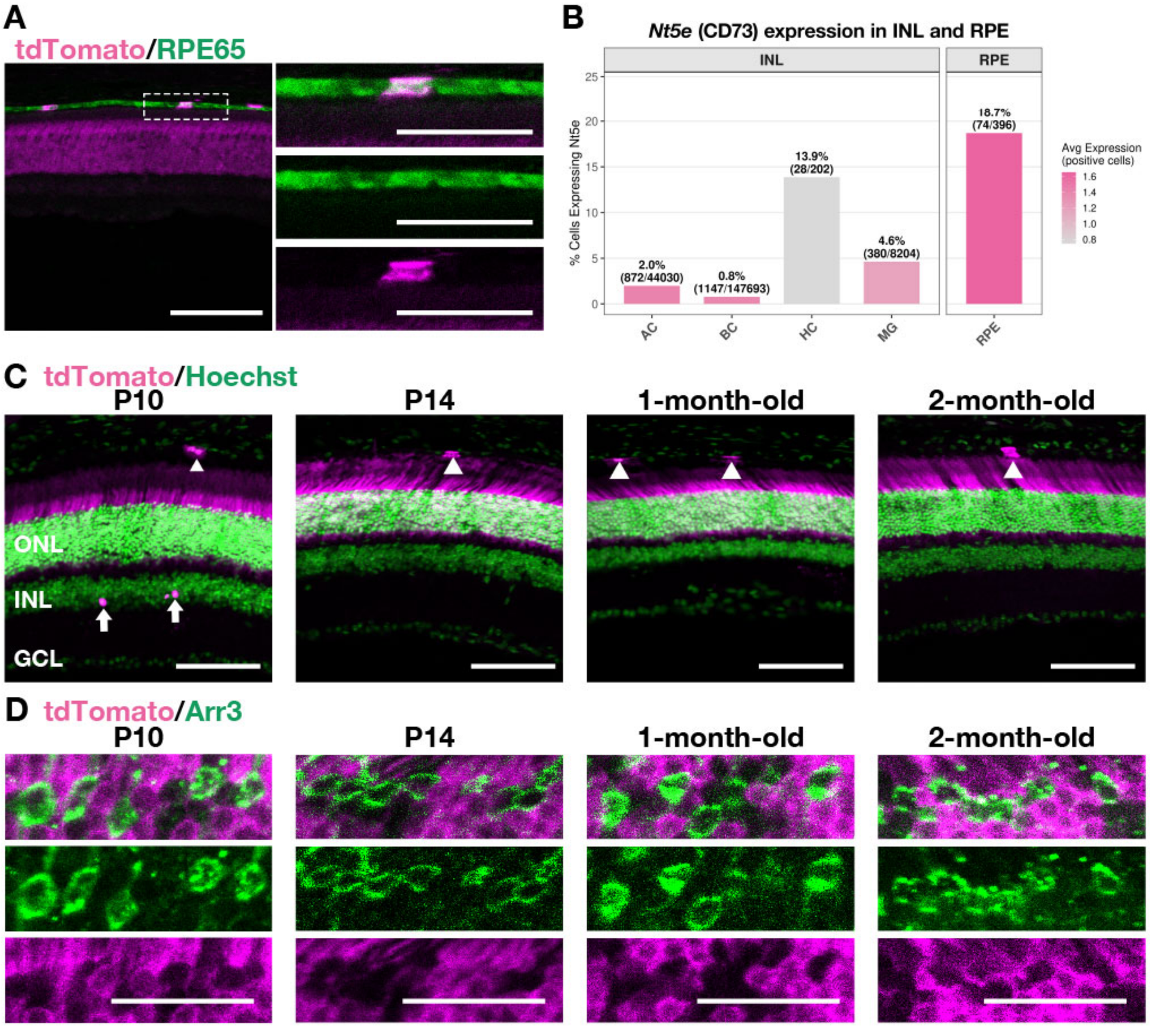

Fig. S6. CD73 expression in retinal pigment epithelium, inner nuclear layer, and rods during retinal maturation. (A) Immunostaining of retinal sections from 3-month-old *CD73-CreER^{T2}* mice two weeks after tamoxifen injection. The left shows a low-magnification view of the entire retina, with RPE65 (green) labeling the retinal pigment epithelium (RPE). tdTomato^{+} cells (magenta) are visible in the RPE layer. The right shows higher-magnification views of the white dashed area in left. The top is the merged image, the middle is RPE65 alone, and the bottom is tdTomato alone. (B) Adult scRNA-seq. Bar plots show the percentage of cells expressing *Nt5e* (CD73) and the average mRNA expression level in inner nuclear layer (INL) cell types (amacrine cells (AC), Müller glia (MG), and horizontal cells (HC)) and in the RPE. (C) Distinct sets of *CD73-CreER^{T2}* mice were each treated with tamoxifen at P10, P14, 1 month, or 2 months of age, and all were examined at 3 months of age. Nuclei are labeled with Hoechst (green), and tdTomato is shown in magenta. Arrowheads indicate tdTomato^{+} cells in the RPE, arrows mark tdTomato^{+} cells in the INL. (D) Higher-magnification views of the outer portion of the ONL (cone-rich region), in the same groups described in (A). Arr3 (cone arrestin, green) labels cone photoreceptors; tdTomato is shown in magenta. The top shows merged i mages, the middle shows Arr3 alone, and the bottom shows tdTomato alone. Scale bars: 100 µm (A, left; C); 25 µm (A, right; D). Abbreviation: GCL, ganglion cell layer; ONL, outer nuclear layer.

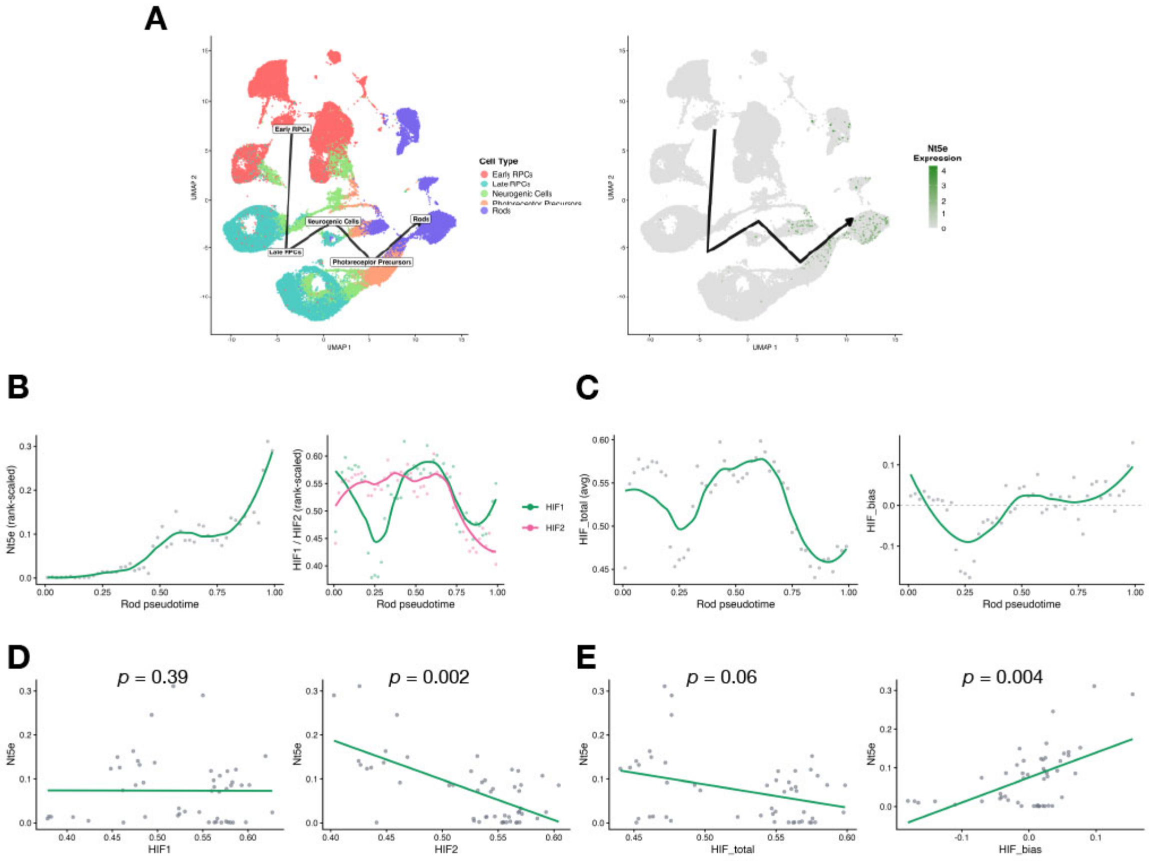

Fig. S7. Association of *Nt5e* (CD73) mRNA with HIF-1/HIF-2 signature scores along the rod-lineage pseudotime. (A) Pseudotime trajectory of the rod lineage visualized in UMAP space, spanning early retinal progenitor cells (early RPCs), late RPCs, neurogenic RPCs, photoreceptor precursors and rods; cell-type identities follow the original publication (left). Cell-wise *Nt5e* normalized expression plotted along pseudotime (right). (B) Smoothed *Nt5e* trend across pseudotime (left). Smoothed trends of HIF-1 and HIF-2 signature scores across pseudotime (right). (C) Smoothed trend of the combined HIF signature (HIF-total; defined as the average of HIF-1 and HIF-2 scores) (left) and the HIF-bias score (reflecting the relative dominance of HIF-1 over HIF-2) (right). In the HIF-bias score, positive values indicate a relative HIF-1 bias. (D) Correlation of *Nt5e* with HIF-1 (left) and HIF-2 (right) signature scores. Spearman's correlation coefficients (ρ) and two-sided p values were calculated using binned average expression values (n = 50 bins) (ρ = -0.12 and -0.47; FDR-adjusted p values are shown on the plots). (E) Correlation of *Nt5e* with the combined HIF signature (left) and the HIF-bias score (right) (ρ = -0.28 and 0.43; FDR-adjusted p values are shown on the plots). Abbreviation: FDR, false discovery rate.

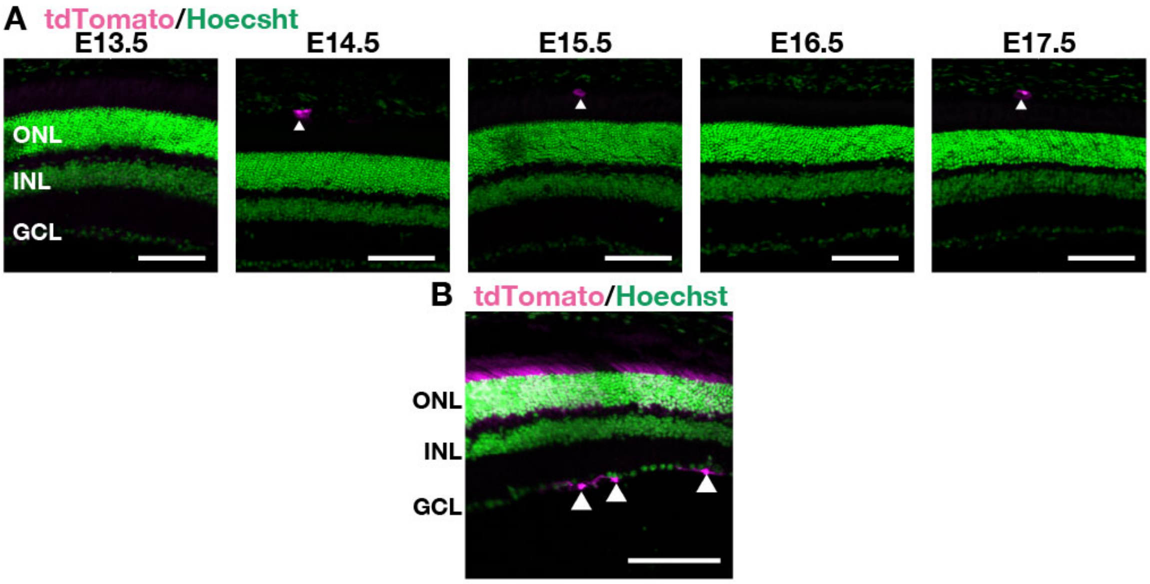

Fig. S8. *CD73-CreER^{T2}* lineage tracing demonstrates CD73^{+} cells in the RPE and RNFL during early developmental stages. (A) Distinct sets of *CD73-CreER^{T2}* mice were each treated with tamoxifen at E13.5, E14.5, E15.5, E16.5, and E17.5, and all were examined at 3 months of age. Nuclei are labeled with Hoechst (green), and tdTomato (magenta) is visible in the RPE (arrowheads). (B) Immunostaining of retinal sections from a *CD73-CreER^{T2}* mouse treated with tamoxifen at P3 and analyzed at 3 months of age. Nuclei are labeled with Hoechst (green), and tdTomato is shown in magenta. Arrowheads indicate tdTomato^{+} cells, presumed to reside in the RNFL immediately vitreal to the GCL. Scale bars: 100 µm (A; B). Abbreviation: GCL, ganglion cell layer; INL, inner nuclear layer; ONL, outer nuclear layer; RNFL, retinal nerve fiber layer; RPE, retinal pigment epithelium.

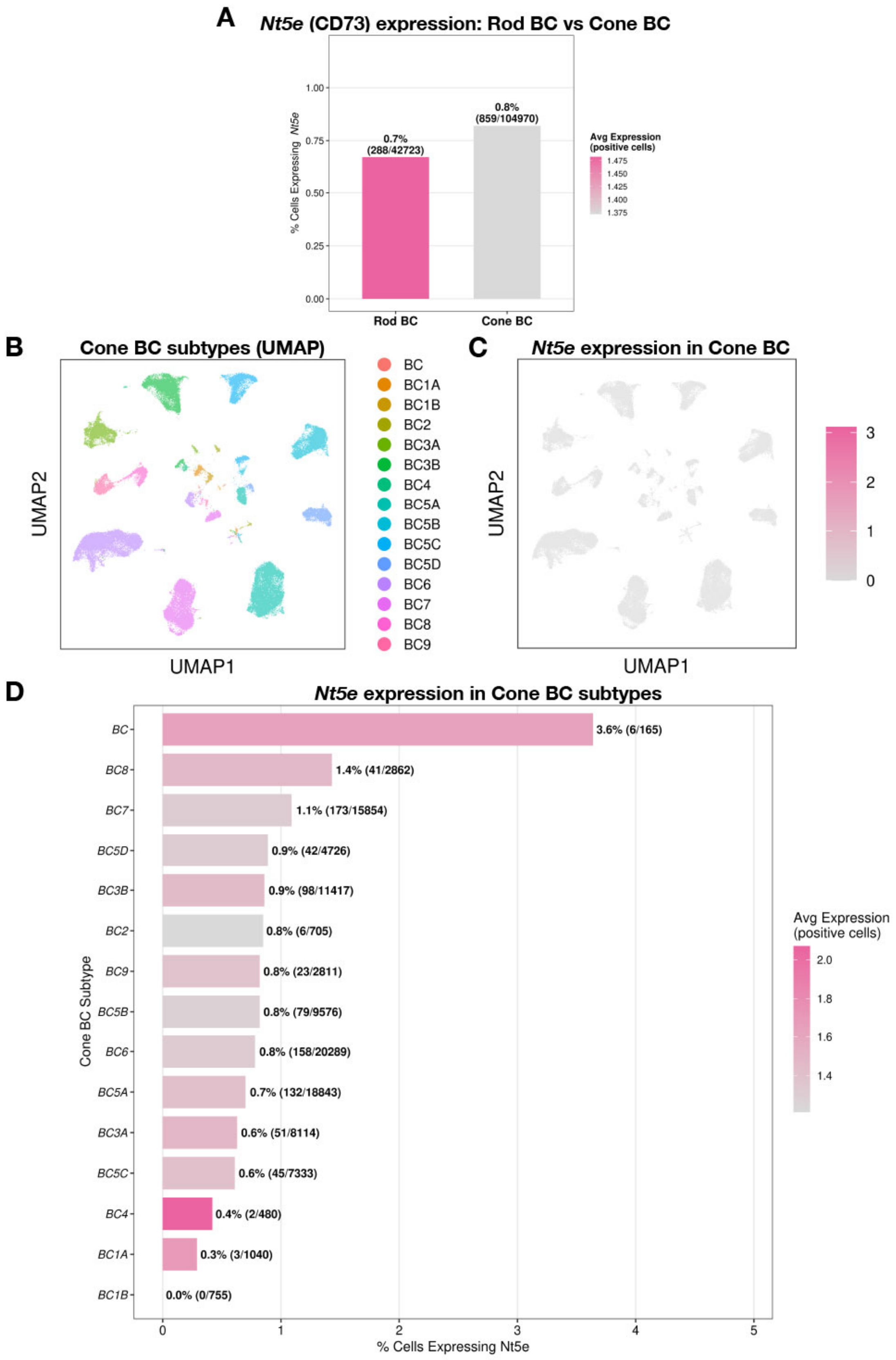

Fig. S9. mRNA-level *Nt5e* (CD73) expression in cone bipolar cell subtypes. (A) Bar plots show *Nt5e* expression in rod and cone bipolar cells (BC). A cell was counted as positive for

Development • Supplementary information

Nt5e when the log-normalized expression value was greater than zero (corresponding to at least one detected UMI). All annotations follow the source study. (B) UMAP plots show cone bipolar cell subtypes. Cell-type identities follow the original publication. (C) Feature plots show *Nt5e* expression. (D) Bar plots show *Nt5e* expression in cone BC subtype. A cell was counted as *Nt5e*-positive when the log-normalized expression value was greater than zero (corresponding to at least one detected UMI). All annotations follow the source study.

Development • Supplementary information

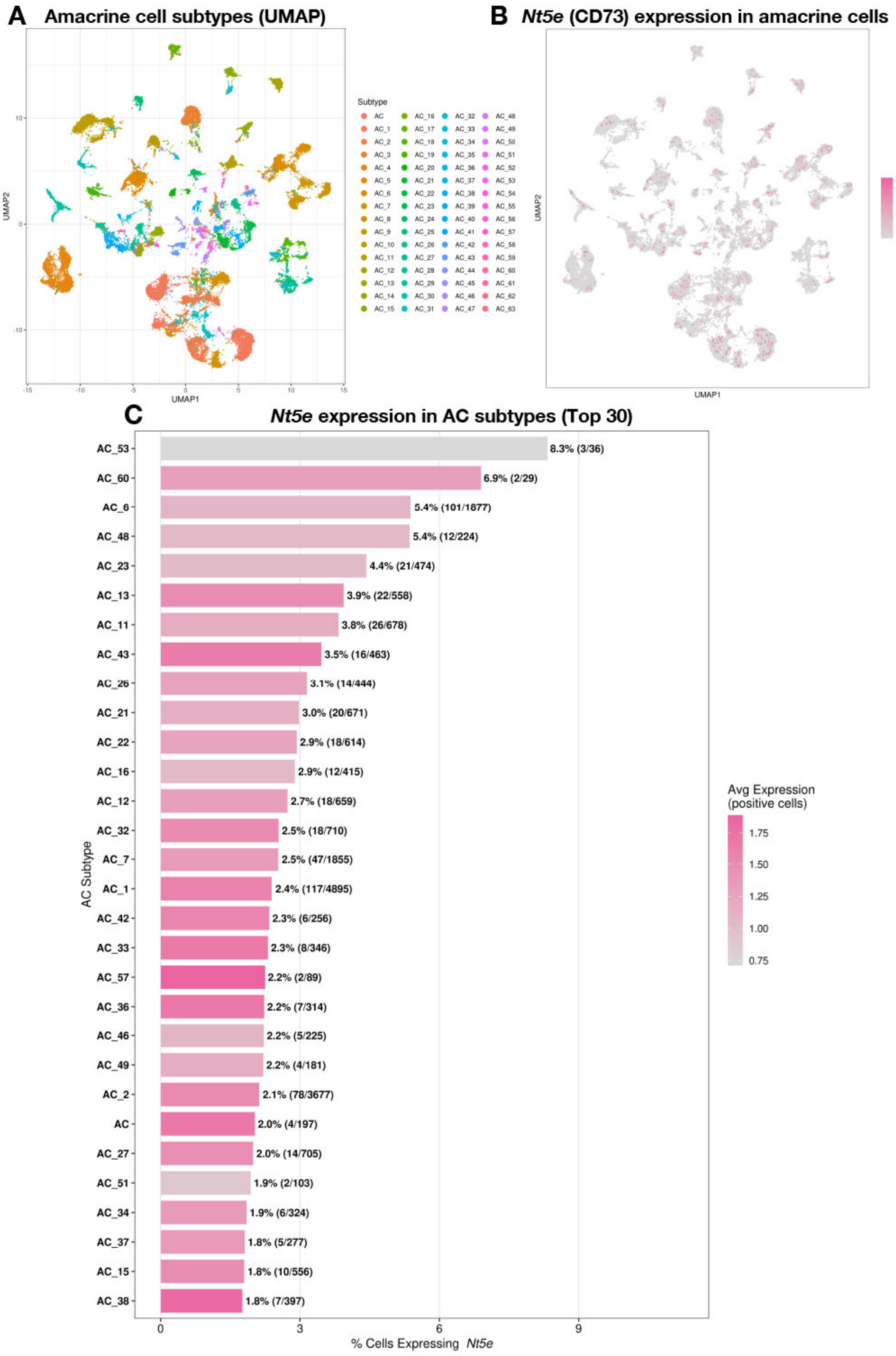

Development • Supplementary information

Fig. S10. mRNA-level*Nt5e* (CD73) expression in amacrine cell subtypes. (A) UMAP plot shows amacrine cell (AC) subtypes. Cell-type identities follow the original publication. (B) Feature plot shows *Nt5e* expression. (C) Bar plots show *Nt5e* expression in amacrine cell subtypes (top 30 cell types). A cell was counted as *Nt5e*-positive when the log-normalized expression value was greater than zero (corresponding to at least one detected UMI). All annotations follow the source study.

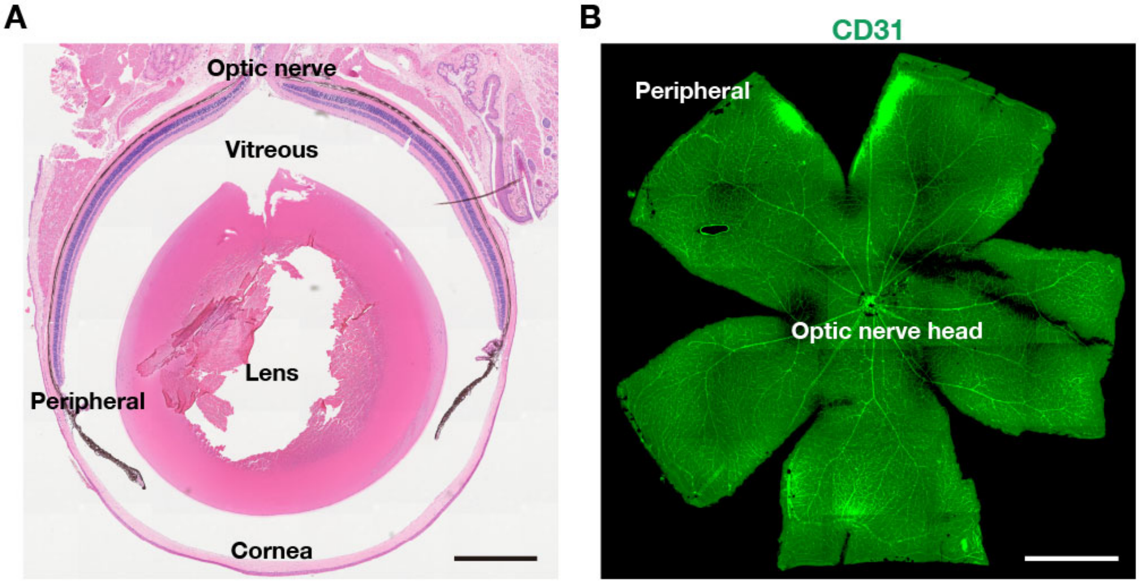

Fig. S11. Representative images illustrating the anatomy of the adult eye. (A) A hematoxylin- and eosin-stained (H&E) cross-section of the eye from a 3-month-old mouse, showing the spatial characteristics of each compartment. (B) A whole-mount retina from a 3- month-old mouse immunostained for CD31 (a vascular endothelial marker) shown in green. Scale bars: 500 µm (A); 1000 µm (B).

Development • Supplementary information

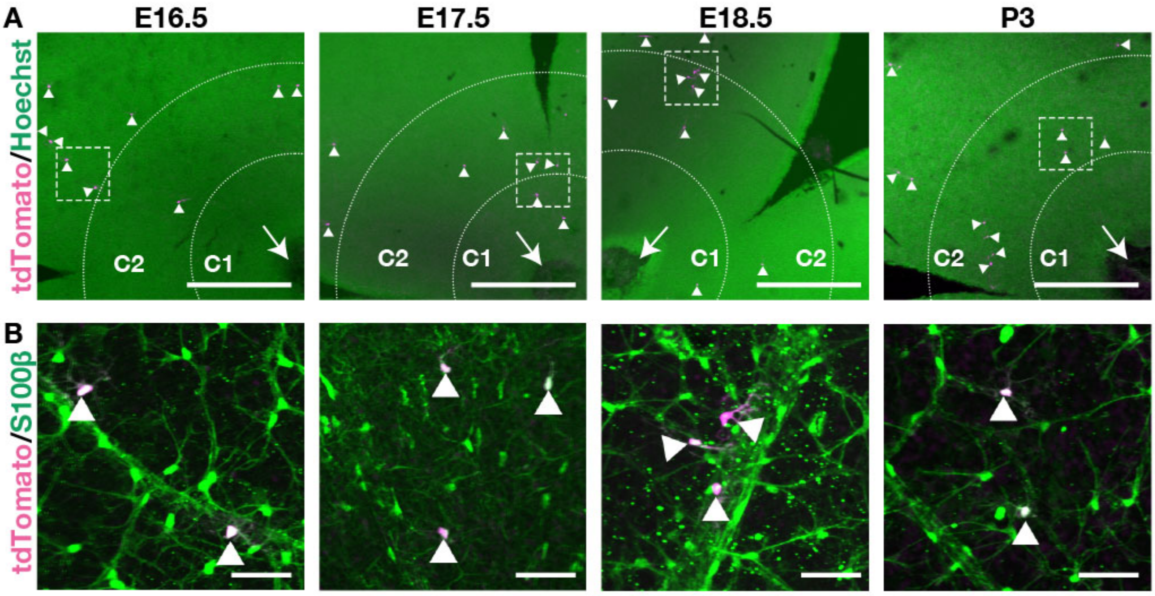

Fig. S12. Descendants of CD73-CreER^{T2+} cells in the astrocyte lineage in the central retina. (A) Representative whole-mount retinal images from *CD73-CreER^{T2};tdTomato* mice treated with tamoxifen at E16.5, E17.5, E18.5, or P3. Shown are maximum-intensity projection images spanning from the INL to the RNFL, excluding the ONL, giving an overview of the central retina in each tamoxifen treatment group. Nuclei are labeled with Hoechst (green), and tdTomato is shown in magenta. The arrow indicates the optic nerve head. (B) Higher-magnification views of the white dashed boxes in (A). The astrocyte marker S100β is labeled in green, and tdTomato is shown in magenta. Arrowheads indicate tdTomato and S100β double-positive cells. All samples were collected at 3 months of age. Scale bars: 500 µm (A); 50 µm (B).

Development • Supplementary information

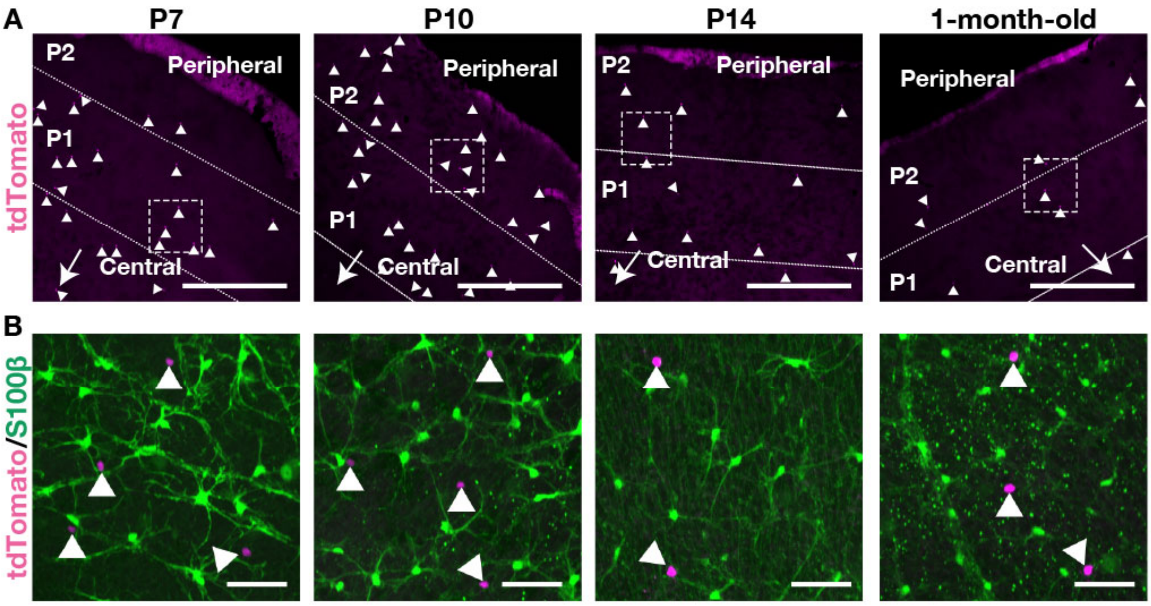

Fig. S13. The distribution pattern of descendants of CD73-CreER^{T2+} cells in the INL cells of the peripheral retina. (A) Representative wholemount retinal images from *CD73-CreER^{T2};tdTomato* mice treated with tamoxifen at P7, P10, P14, or 1 month of age. Shown are maximum-intensity projection images from the INL to the RNFL, excluding the ONL, providing an overview of the peripheral retina for each treatment group. tdTomato is shown in magenta. Arrowheads indicate tdTomato^{+} cells in the INL. ‘Peripheral’ marks the retinal
144 periphery; the arrow labeled ‘Central’ indicates the direction toward the optic nerve head. (B) Higher-magnification views of the white dashed boxes in (A). The astrocyte marker S100β is labeled in green, and tdTomato is shown in magenta. These images confirm that the tdTomato^{+} cells in (A) reside in the INL and lack S100β expression. All samples were collected at 3 months of age. Scale bars: 500 µm (A); 50 µm (B).

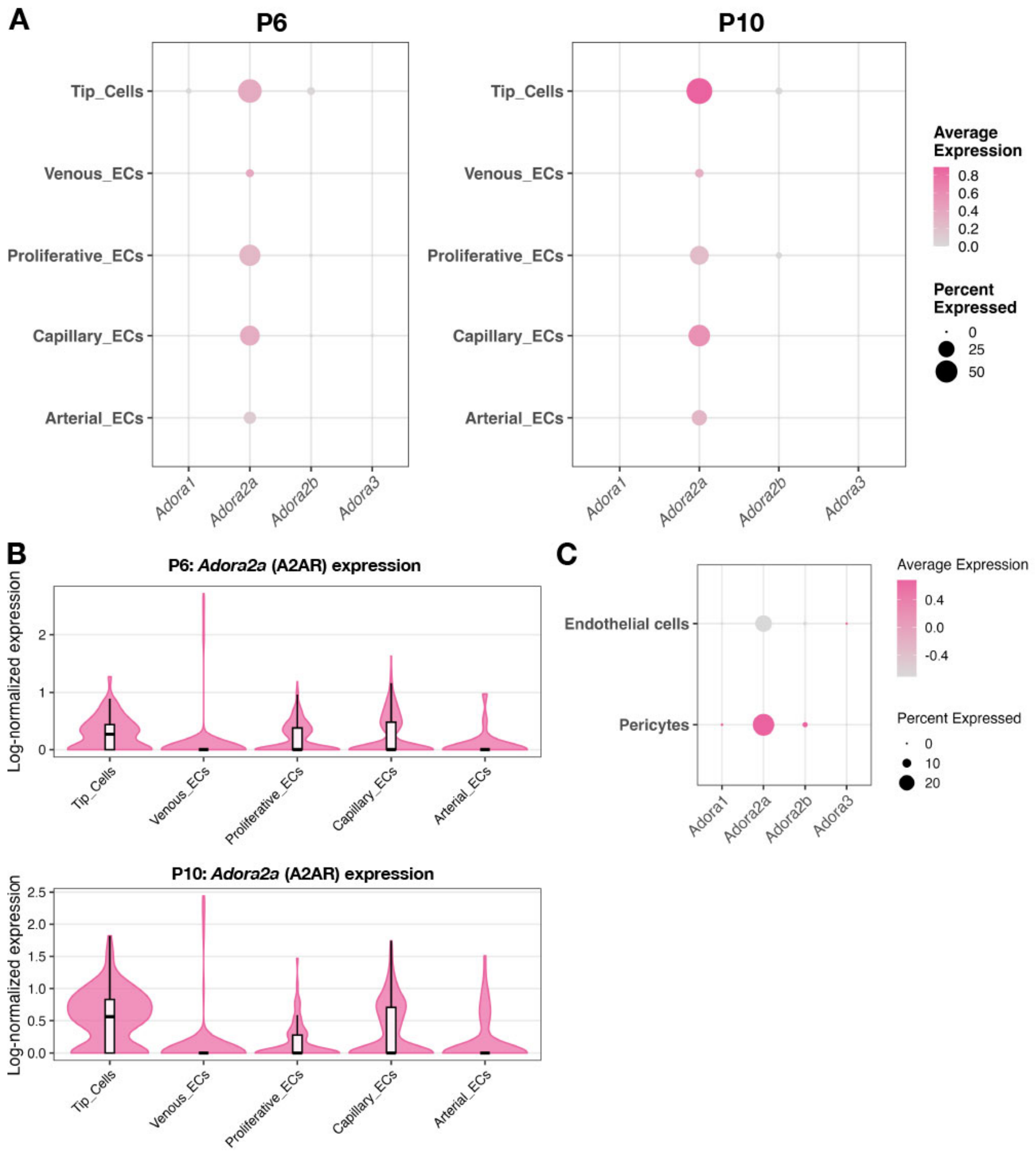

Fig. S14. Adenosine receptor expression in the retinal vasculature (endothelial cells and pericytes). (A) Developmental scRNA-seq (P6 and P10; CD31-enriched preparations). Dot plots show the fraction and average mRNA expression of *Adora1* (A1R), *Adora2a* (A2AR), *Adora2b* (A2BR), and *Adora3* (A3R) in endothelial cells at P6 (left) and P10 (right). Endothelial subcluster identities follow the original publication. (B) Violin plots of *Adora2a* expression in endothelial subclusters at P6 (top) and P10 (bottom). (C) Adult scRNA-seq. Dot plots show the same genes in pericytes, and endothelial cells as annotated in the source study. Endothelial cells were not further subclustered in this dataset. Abbreviation: EC, endothelial cell.

Development • Supplementary information

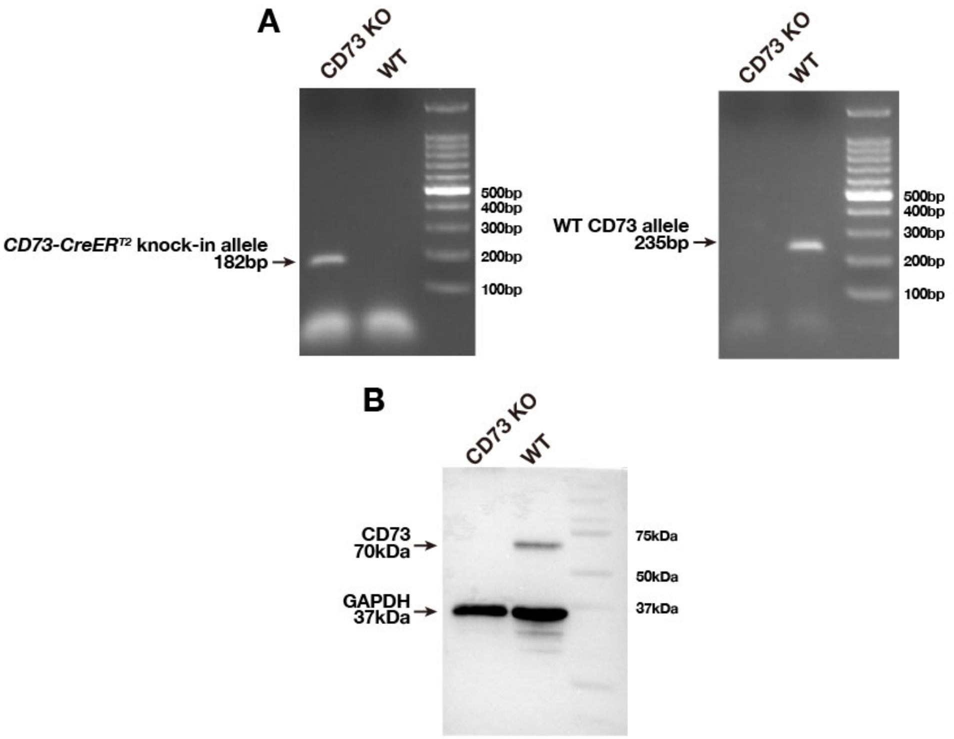

Fig. S15. Confirmation of CD73 expression deficiency in CD73 KO mice. (A) Representative genotyping results. The left shows the *CD73-CreER^{T2}* knock-in allele (primer product, 182 bp), and the right shows WT CD73 allele (primer product, 235 bp). In the left panel, the CD73 KO displays a band corresponding to the *CD73-CreER^{T2}* knock-in allele, whereas the WT does not. In the right, the CD73 KO lacks the band for the WT CD73 allele, while the WT displays that band. (B) Representative immunoblot. The expected molecular weights of CD73 (~70 kDa) and GAPDH (~37 kDa) are indicated. Both CD73 KO and WT samples show a GAPDH band at ~37 kDa, but only the WT shows a CD73 band at ~70 kDa; the CD73 KO lacks this band, confirming the deficiency of CD73 expression. Abbreviations: bp, base pairs; KO, knock-out; WT, wild-type.

Development • Supplementary information

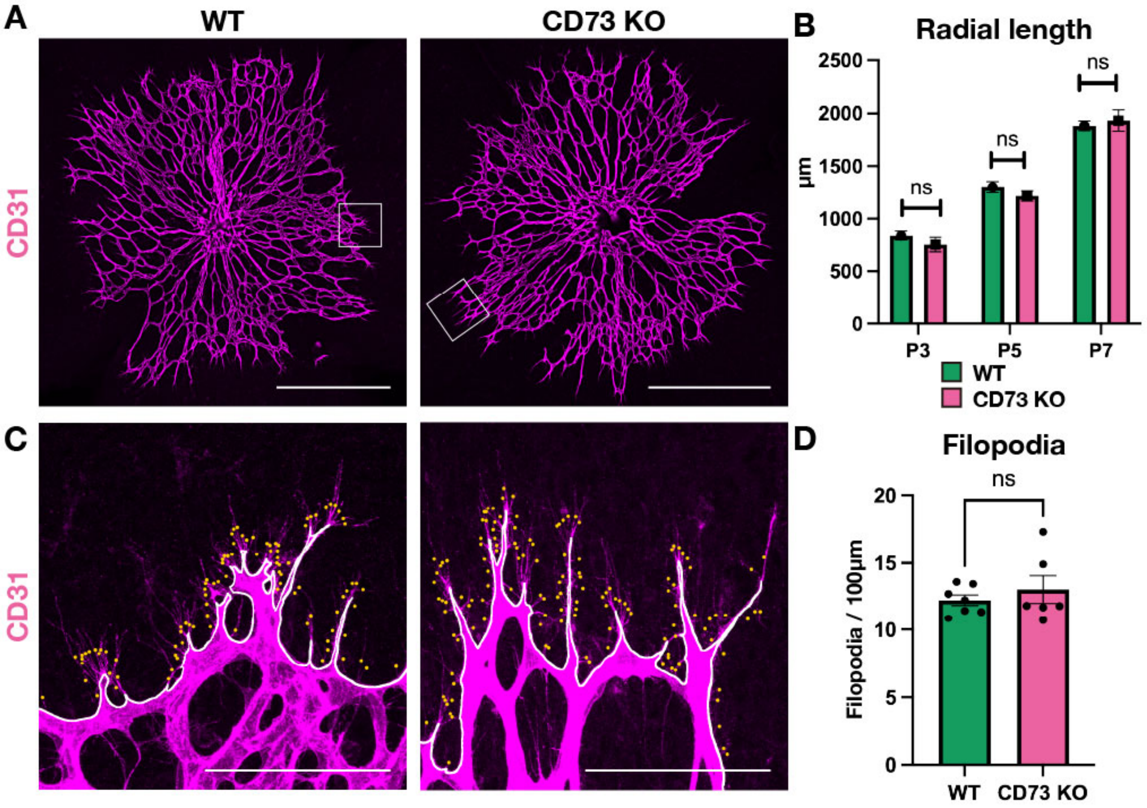

Fig. S16. Early postnatal superficial plexus in CD73 KO mice (P3–P7). (A) Representative whole-mount retinas at postnatal day 3 (P3) from WT (left) and CD73 KO (right). CD31 (vascular endothelium), magenta. (B) Radial outgrowth length from the optic nerve head at P3, P5, and P7. Data are shown as mean ± s.e.m. (C) High-magnification views of the vascular front at P3. The white line indicates the front edge; filopodia are labeled in yellow. (D) Quantification of filopodia at P3. Counts were normalized to the length of the vascular front (number per 100 µm). Data are presented as mean ± s.e.m., with each dot representing an individual sample. Statistical significance was assessed using unpaired two-tailed Student's *t*-test (B, D). Sample sizes: n = 7 WT, n = 6 CD73 KO at P3; n = 4 WT, n = 5 CD73 KO at P5; n = 5 WT, n = 3 CD73 KO at P7. Scale bars: 500 µm (A); 100 µm (C).

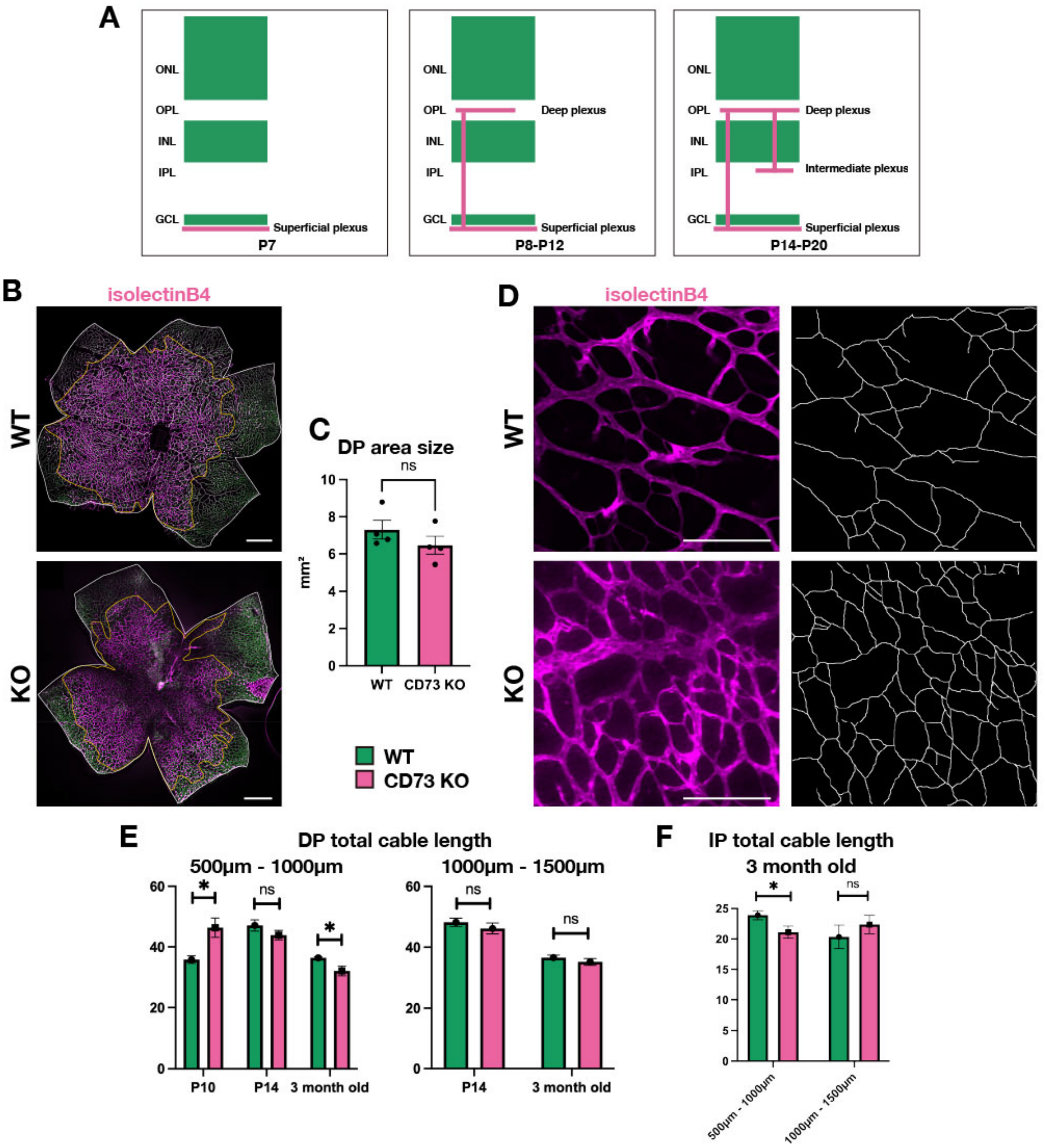

Fig. S17. Development of the deep and intermediate plexuses in CD73 KO mice. (A) Schematic of retinal vascular development at P7 (left), P8–P12 (center), and P14–P20 (right). (B) Representative whole-mount retinas at P10 from WT (top) and CD73 KO (bottom). The superficial plexus is green. The deep plexus (DP) is magenta. White and yellow outlines mark the boundaries of the superficial and deep plexuses, respectively. (C) Quantification of the deep plexus (DP) at P10 (n = 4 WT, n = 4 CD73 KO). Data are shown as mean ± s.e.m., with each dot representing an individual sample. (D) Left: higher-magnification views of the deep plexus at P10 labeled with isolectin B4 (IB4, magenta). Right: representative Simple Neurite Tracer (SNT) tracings in FIJI used for morphometry. (E) SNT-derived total cable length in the deep plexus (DP) within the 500–1000 µm annulus (left) and the 1000–1500 µm annulus (right) from the optic nerve head. Data are shown as mean ± s.e.m. Sample sizes: n = 6 WT, n

Development • Supplementary information

= 5 CD73 KO at P10; n = 4 WT, n = 5 CD73 KO at P14; n = 7 WT, n = 8 CD73 KO at 3 months of age. (F) SNT-derived total cable length in the intermediate plexus (IP) within the 500–1000 µm and 1000–1500 µm annuli from the optic nerve head at 3 months of age (n = 7 WT, n = 8 CD73 KO). Data are shown as mean ± s.e.m. Statistical significance was assessed using unpaired two-tailed Student's *t*-test (* $p < 0.05$). Quantified regions are illustrated in Fig. S16. Scale bars: 500 µm (B); 100 µm (C).

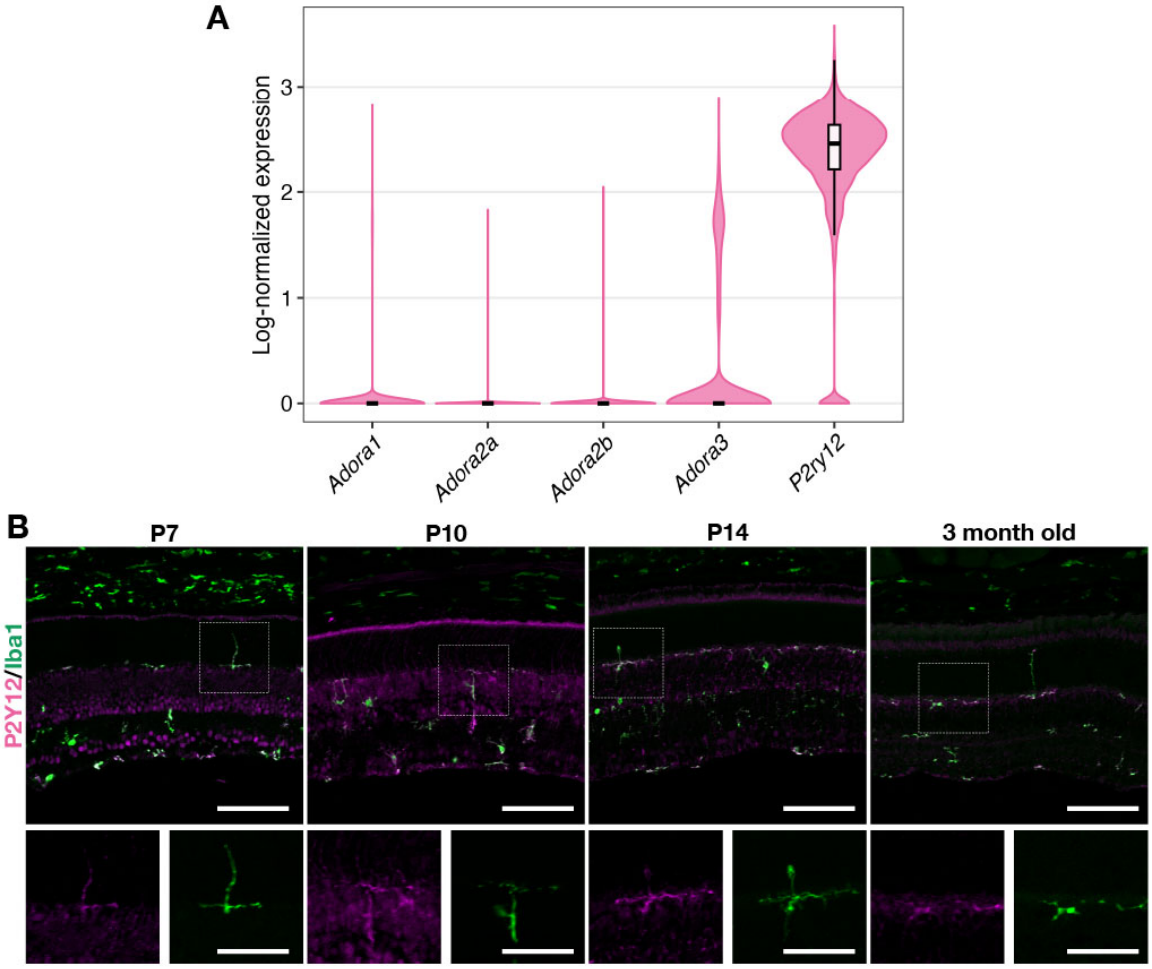

Fig. S18. Adenosine receptors and P2ry12 in microglia. (A) Adult scRNA-seq. Violin plots show mRNA expression of *Adora1* (A1R), *Adora2a* (A2AR), *Adora2b* (A2BR), *Adora3* (A3R) and *P2ry12* (P2Y12) in microglia. Microglia annotation follows the source study. (B) Transverse retinal sections from CD73-EGFP mice at P7, P10, P14 and 3 months of age. For each field, the top row shows P2Y12 (magenta) with Iba1 (green), and the bottom row shows Iba1 alone (left) and P2Y12 alone (right). Scale bars: 100 µm (B, top); 50 µm (B, bottom).

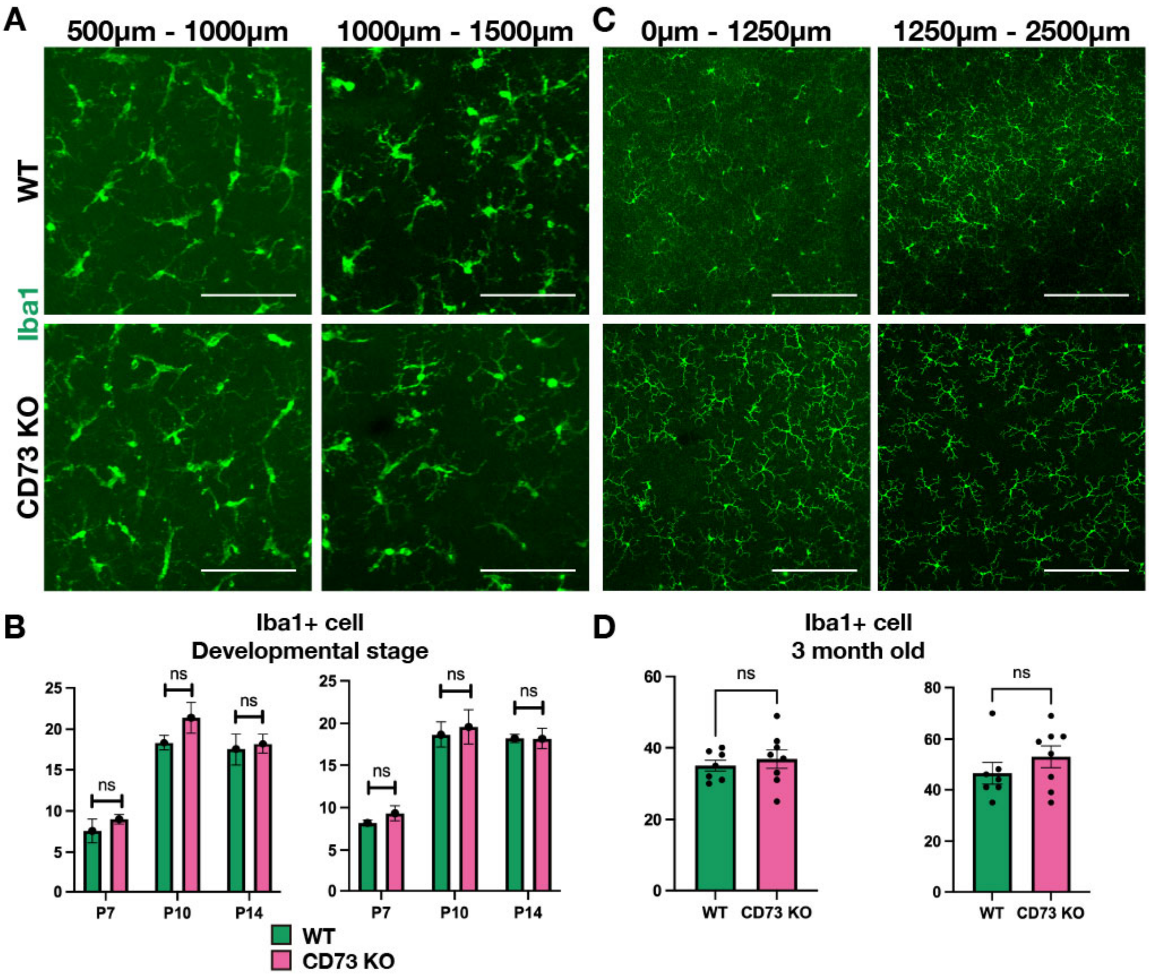

Fig. S19. Microglial quantification in the OPL of CD73 KO mice. (A) Representative whole-mount outer plexiform layer (OPL) images at P10 from WT (top) and CD73 KO (bottom) within the 500–1000 µm (left) and 1000–1500 µm (right) annuli from the optic nerve head (ONH). Iba1 (microglial marker), green. (B) Quantification of $Iba1^+$ microglia at P7, P10, and P14 in the 500–1000 µm (left) and 1000–1500 µm (right) annuli from the ONH. Data are shown as mean ± s.e.m. (C) Representative whole-mount OPL images at 3 months of age from WT (top) and CD73 KO (bottom) within the 0–1250 µm (left) and 1250–2500 µm (right) annuli from the ONH. Iba1 (microglial marker), green. (D) Quantification of $Iba1^+$ microglia at 3 months of age in the 0–1250 µm (left) and 1250–2500 µm (right) annuli from the ONH. Data are shown as mean ± s.e.m. Sample sizes: n = 5 WT, n = 3 CD73 KO at P7; n = 6 WT, n = 5 CD73 KO at P10; n = 4 WT, n = 5 CD73 KO at P14; n = 7 WT, n = 8 CD73 KO at 3 months of age. Statistical significance was assessed using unpaired two-tailed Student's *t*-test. Scale bars: 100 µm (A); 200 µm (C).

Development • Supplementary information

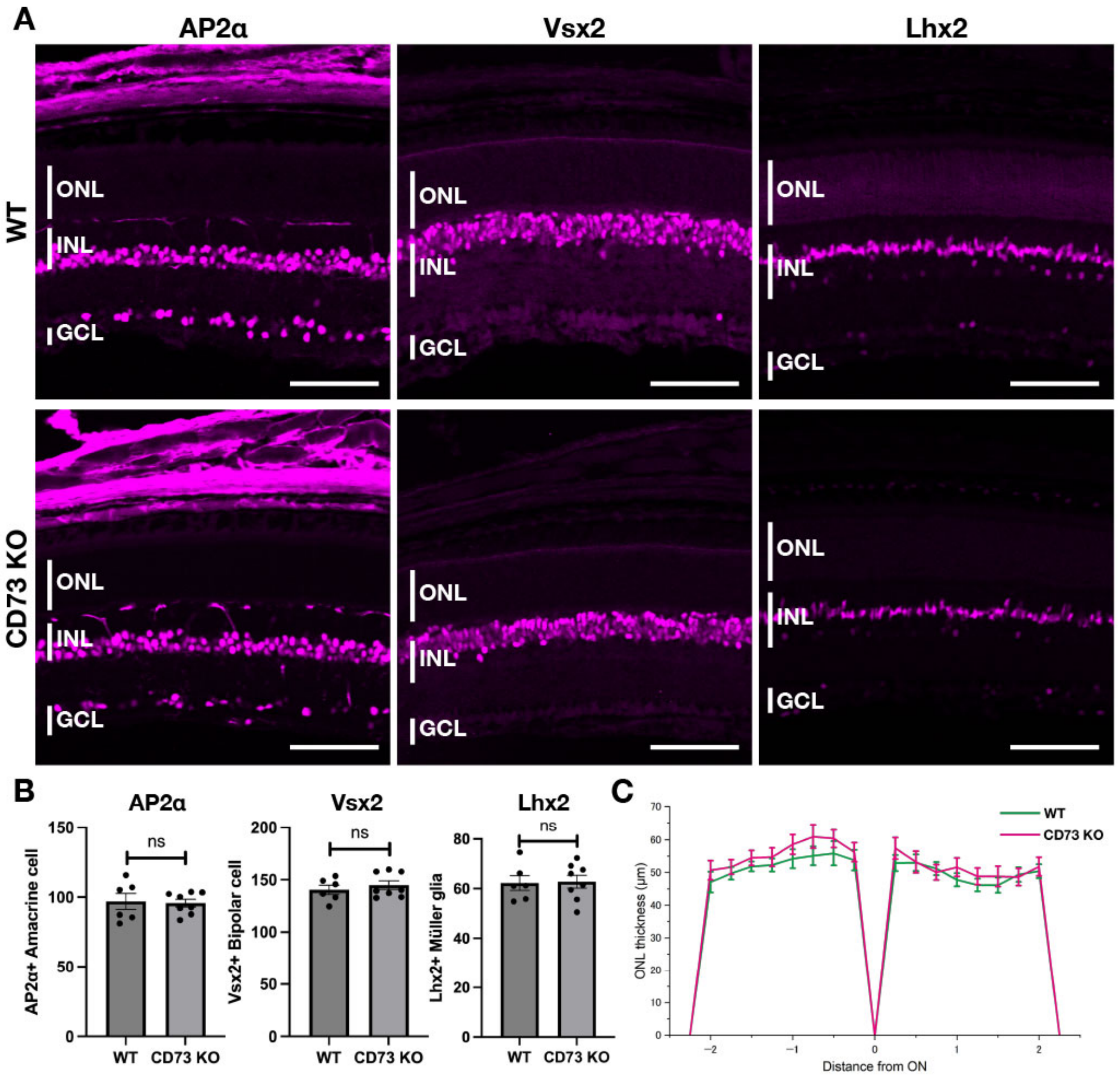

Fig. S20. CD73 KO mice show no abnormalities in retinal cell differentiation under physiological conditions. (A) Representative retinal sections from WT and CD73 KO mice at postnatal day 14 (P14). Sections were immunostained for AP2α (amacrine cells), Vsx2 (bipolar cells), and Lhx2 (Müller glia). (B) Quantification of AP2α^{+}, Vsx2^{+}, and Lhx2^{+} cells in the INL. Data are shown as mean ± s.e.m., with each dot representing an individual sample (n = 6 WT, n = 8 CD73 KO). Statistical significance was determined using unpaired two- tailed Student's *t*-test. (C) Measurement of ONL thickness (in µm) along the superior (0–2.25 mm) and inferior (-2.25–0 mm) hemiretina in WT and KO mice. Data are shown as mean ± s.e.m. (n = 9 WT, n = 9 CD73 KO). All statistical analyses were performed in GraphPad Prism v10 (GraphPad Software). Scale bars: 100 µm (A).

Development • Supplementary information

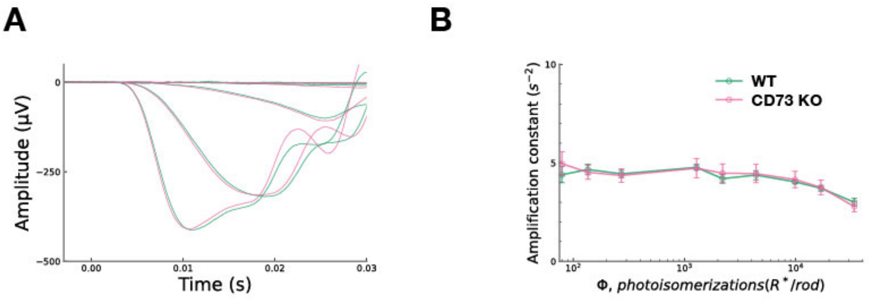

Fig. S21. Activation processes of the rod phototransduction cascade. (A) The average a-wave of WT (green, n = 10) and CD73 KO (magenta, n = 10) mice. (B) Plot of the amplification constant of rod photoreceptors as a function of the number of the rhodopsin photoisomerizations. Data are shown as mean ± s.e.m. (n = 10 WT, n = 10 CD73 KO). Statistical significance was assessed using unpaired two-tailed Student's *t*-test (*$p < 0.05$). All ERG recordings were performed in 3-month-old mice.

Development • Supplementary information

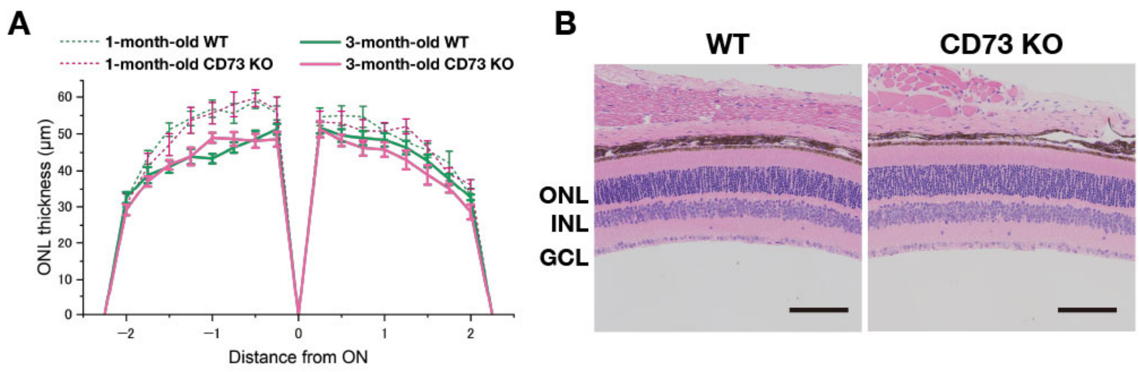

Fig. S22. ONL thickness profiles in the light-exposed region during postnatal growth. (A) ONL thickness (µm) measured along the superior (0–2.25 mm) and inferior (-2.25–0 mm) hemiretina in WT (green) and CD73 KO (magenta) mice at 1-month-old (dashed lines) and 3-month-old (solid lines). Data are presented as mean ± s.e.m. (1-month-old: n = 11 WT, n = 7; CD73 KO; 3-month-old: n = 10; WT n = 9 KO). Statistical significance was assessed using unpaired two-tailed Student's *t*-test at each eccentricity with Benjamini–Hochberg false discovery rate (FDR) correction for multiple comparisons across retinal positions at each age; no position reached significance after FDR correction (all FDR-adjusted $p > 0.05$). (B) Representative H&E-stained sections of WT and CD73 KO retinas at 3 months of age. Retinal sections were cut along the vertical meridian at the level of the optic nerve; the image shown is located approximately 1000 µm inferior to the optic nerve. Scale bars: 100 µm (B). Abbreviation: ONL, outer nuclear layer.

Development • Supplementary information

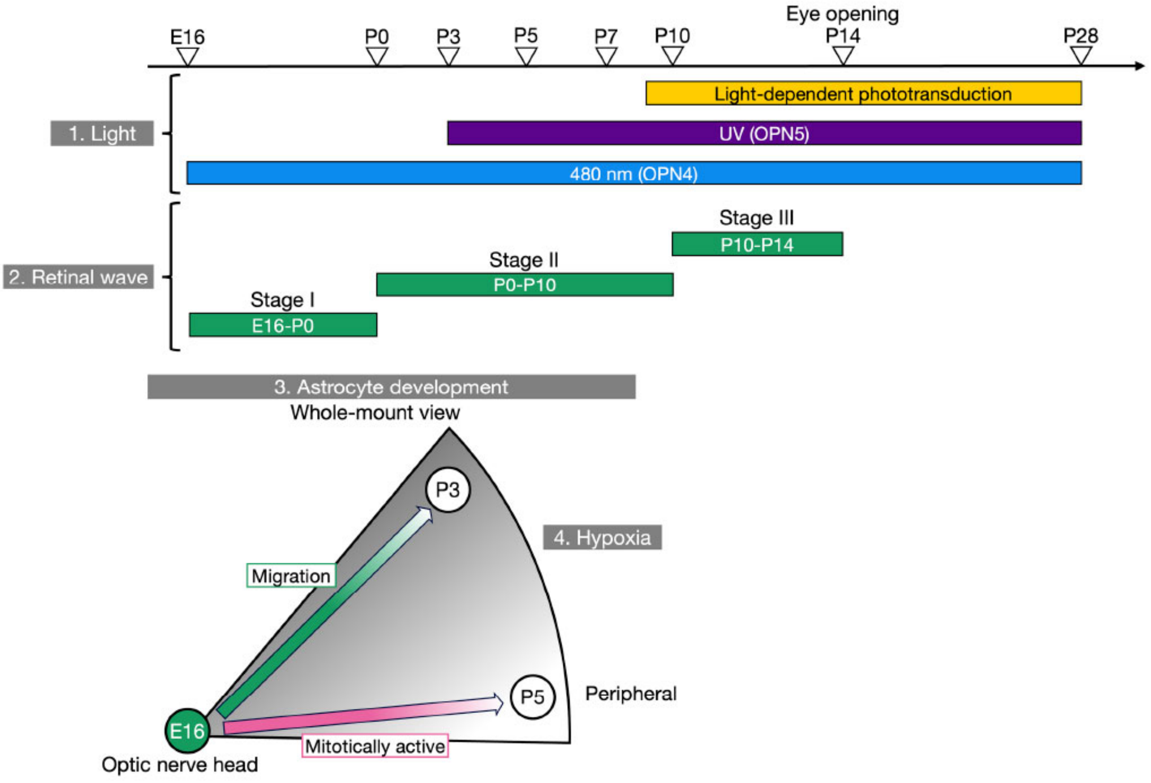

Fig. S23. Environmental stimuli and developmental milestones. (1) Light: OPN4 and OPN5 signaling begins at E16 and P3, respectively. Rod phototransduction begins around P8. (2) Retinal waves: Retinal waves occur in three phases: Stage I (E16–P0), Stage II (P0–P10), and Stage III (P10–P14). (3) Astrocytes: Astrocyte progenitors migrate from the optic nerve head toward the periphery and remain mitotically active until around P5. (4) Hypoxia: Hypoxia gradually resolves concomitantly with retinal vascular development.

Table S1. Antibodies and chemicals used in this study.

Reagent type	Designation	Source	Identifiers	Additional information
Antibody	Sheep anti-Chx10	Exalpha Biologicals	Cat# X1179P; RRID: AB_2889828	IF (1:200)
Antibody	Rabbit anti-Cone Arrestin	Millipore	Cat# AB15282; RRID: AB_1163387	IF (1:500)
Antibody	Rabbit anti-Pax2	BioLegend	Cat# 901002; RRID: AB_2565001	IF (1:500)
Antibody	Rabbit anti-RPE65	Abcam	Cat# ab231782; RRID: AB_2922412	IF (1:500)
Antibody	Mouse anti-AP2 α (clone 3B5)	Santa Cruz Biotechnology	Cat# sc-12726; RRID: AB_3676423	IF (1:500)
Antibody	Rabbit anti-Lhx2	Abcam	Cat# ab184337; RRID: AB_2916270	IF (1:500)
Antibody	Rat anti-CD31 (clone MEC13.3)	BD Biosciences	Cat# 550274; RRID: AB_393571	IF (1:250)
Antibody	Rabbit anti-S100 β	Abcam	Cat# ab52642; RRID: AB_882426	IF (1:500)
Antibody	Goat anti-Iba1	Abcam	Cat# ab5076; RRID: AB_2224402	IF (1:500)
Antibody	Rabbit anti-Iba1	FUJIFILM Wako	Cat# 019-19741; RRID: AB_839504	IF (1:500)
Antibody	Rabbit anti-P2Y12	AnaSpec	Cat# AS-55043A; RRID: AB_2298886	IF (1:500)
Antibody	Donkey anti-Rabbit Alexa Fluor 488 AffiniPure IgG (H+L)	Jackson ImmunoResearch	Cat# 711-545-152; RRID: AB_2313584	IF (1:1000)
Antibody	Donkey anti-Rabbit Alexa Fluor Cy3 AffiniPure IgG (H+L)	Jackson ImmunoResearch	Cat# 711-165-152; RRID: AB_2307443	IF (1:1000)
Antibody	Donkey anti-Rabbit Alexa Fluor 647 AffiniPure IgG (H+L)	Jackson ImmunoResearch	Cat# 711-605-152; RRID: AB_2492288	IF (1:1000)
Antibody	Donkey anti-Mouse Alexa Fluor 488 AffiniPure IgG (H+L)	Jackson ImmunoResearch	Cat# 715-545-151; RRID: AB_2341099	IF (1:1000)
Antibody	Donkey anti-rat Alexa Fluor 488 AffiniPure IgG (H+L)	Jackson ImmunoResearch	Cat# 712-545-153; RRID: AB_2340684	IF (1:1000)
Antibody	Donkey anti-Sheep Alexa Fluor 647 Cross-Adsorbed IgG (H+L)	Invitrogen (Thermo Fisher Scientific)	Cat# A21448; RRID: AB_2535865	IF (1:1000)
Chemical	Isolectin GS-IB4, Alexa Fluor 647 Conjugate	Invitrogen	Cat# I32450; RRID: SCR_014365	IF (1:250)

IF, immunofluorescence; RRID, Research Resource Identifier.
